# Hemostatic instantaneous coagulation on echocardiogram: a defining feature of the last heartbeat (a case report)

**DOI:** 10.11604/pamj.2022.42.7.34446

**Published:** 2022-05-05

**Authors:** Tokunbo David Gbadebo, Daniel Antwi-Amoabeng, Ademola Abiose

**Affiliations:** 1 Emory Decatur Hospital, Decatur, Georgia, United States of America,; 2 Christus Ochsner St. Patrick Hospital, Lake Charles, Louisiana, United States of America,; 3 University Hospitals, Case Medical Center, Cleveland, Ohio, United States of America

**Keywords:** Echocardiography, cardiac arrest, spontaneous echo contrast, sinoventricular rhythm, case report

## Abstract

There is a paucity of detailed descriptions of echocardiographic features of the dying heart in the literature. A 64-year-old man on chronic hemodialysis presented with cardiac arrest after missing dialysis for three weeks. He received resuscitation efforts but died while his last heartbeats were fortuitously recorded by echocardiography. Rapid echo image acquisition during pulse check of a cardiopulmonary resuscitation attempt provided a unique opportunity for documenting the echocardiographic features of a dying heart. There was a rapid progressive dense echogenicity first in the left ventricular chamber and subsequently in all other chambers, which coincided with the final heartbeats. There is no prior documentation of this observation in the literature. We hereby illustrate and characterize this observation we term as Hemostatic Instantaneous Coagulation on Echo (HICE). HICE may be the defining feature of the dying heart and may guide the decision to discontinue resuscitation interventions.

## Introduction

Echocardiography is perhaps the single most versatile imaging modality for assessment of the structural and functional state of the heart. In critical situations of hemodynamic instability such as circulatory collapse or shock, it can provide valuable information on elements that impact cardiac function, which can guide patient management. Echocardiography is not commonly done in imminently dying patients, and such images are not easily encountered. In this report, we illustrate and discuss the progressive echocardiographic findings in a dying heart during resuscitative efforts with advanced cardiovascular life support (ACLS) protocol until the last heartbeat was fortuitously recorded.

## Patient and observation

**Figure 1:**
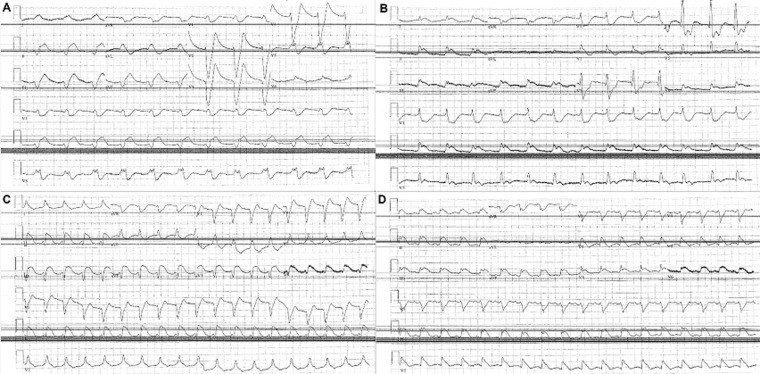
ECG tracings obtained immediately before, and during ACLS interventions; A - C: Sinoventricular rhythm, wide complex tachycardia with absent ST segment, or ST elevation in some leads, and peaked T waves; D: P-waves transiently reappear after the administration of epinephrine, calcium chloride and sodium bicarbonate during ACLS

**Patient information:** a 64-year-old man with past medical history of hypertension and end-stage renal disease on chronic hemodialysis presented with hypothermia, hyperkalemia (8.0 mmol/L), shock and delirium after missing dialysis for 3 weeks. He was noted to be in a wide complex tachycardia that was clinically a pulseless electrical activity (PEA) (Figure 1A). He received defibrillation shocks and advanced cardiovascular life support (ACLS) protocol ensued. He received calcium chloride, sodium bicarbonate, dextrose boluses and insulin. There was transient reappearance of p-waves after the administration of these temporizing therapies for hyperkalemia (Figure 1D), supporting a diagnosis of sinoventricular rhythm. However, the patient required emergent hemodialysis for a more definitive therapy and epinephrine drip for shock.

**Clinical findings:** physical exam was significant for an unresponsive patient with a thready pulse.

**Timeline of current episode:** the patient had previous history of missed hemodialysis with associated hyperkalemia and uremic encephalopathy, but this was the index event of an associated ventricular arrhythmia.

**Diagnostic assessment:** scheduled pulse checks were done during the ACLS resuscitation. Echocardiography images were obtained concurrently during the pulse check periods to rule out other acute structural cardiac anomaly.

**Diagnosis:** pulseless electrical activity with a wide complex tachycardia is considered as a cardiac arrest. Clinical considerations include ventricular tachycardia (VT), accelerated idioventricular rhythm, cardiac tamponade, hypovolemic shock, hyperkalemia, severe acidosis, hypothermia, hypoxia, pulmonary embolism, pneumothorax, and myocardial infarction.

**Figure 2:**
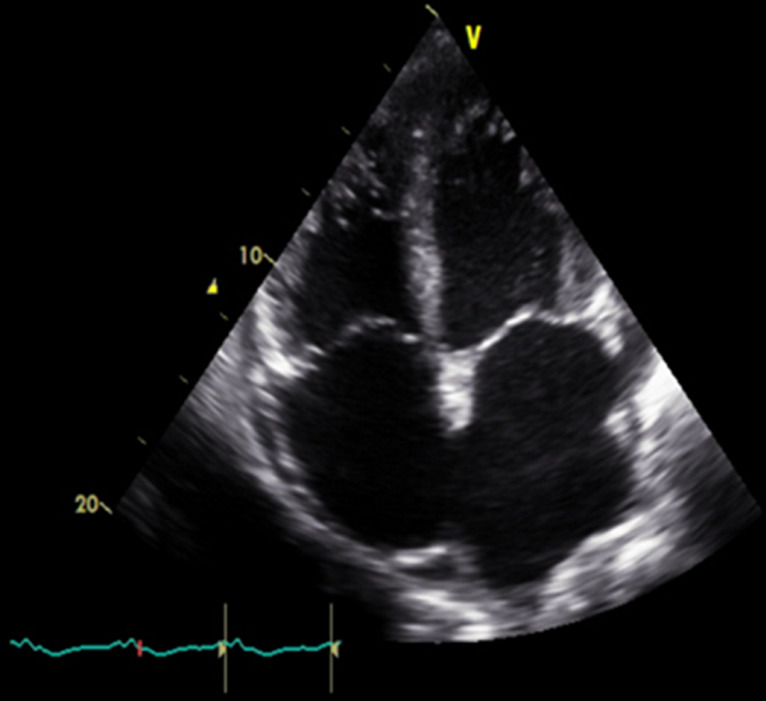
apical 4-chamber view, there is severe dilation of all the chambers and a small amount of pericardial effusion without tamponade physiology, and very subtle granular elements in LV chamber consistent with SEC

**Figure 3:**
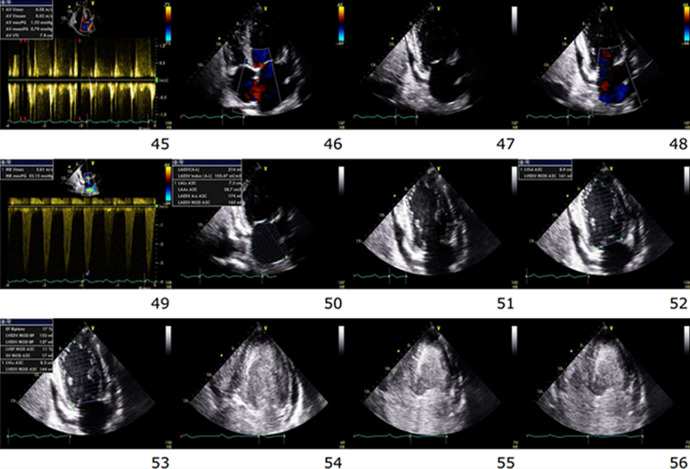
representative frames of echo images obtained during ACLS procedures; image 46 is an apical 4-chamber view showing a small amount of circumferential pericardial effusion present (47 - 48, 51 - 56); there was an abrupt appearance of SE, which begins in frame #54 in the LV, and persists until the final heartbeat

**Figure 4:**
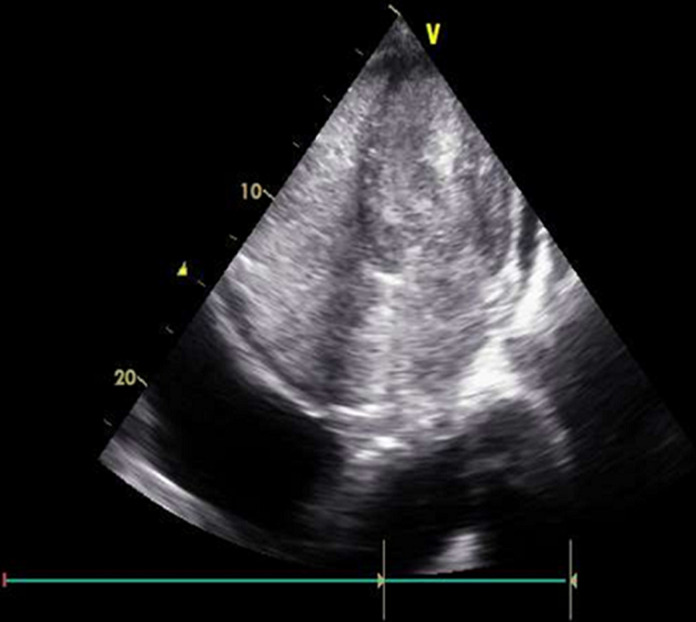
final heartbeat; dense amorphous echogenic material fills the entire LV chamber; this represents hemostatic instantaneous coagulation on echo, HICE, a no-flow state with a complete absence of electrical and mechanical activity

**Therapeutic interventions:** CPR was resumed, and echocardiographic images were obtained intermittently during the pulse check intervals. The first images from standard apical 4-chamber view were recorded about 38 minutes before the patient was pronounced dead. The beginning images showed severe biventricular hypokinesis, LVEF approximately 20%, bi-atrial enlargement, severe mitral regurgitation, and moderate tricuspid regurgitation (Figure 2). There was only a trace of circumferential pericardial effusion and a subtle appearance of granularity of spontaneous echo contrast (SEC) noted in LV chamber only. As time progressed, there was progressive slowing of the heart rate with slightly increasing echogenicity within the LV chamber, and a nominal increase in pericardial effusion. These findings appeared unchanged until long tandem episodes of asystole heralded the last heart beats shortly thereafter. In the last 50 secs to the last heartbeat, continuous echo recording was obtained. We documented a rapid progressive echogenicity and dense thrombus formation in the LV chamber, cessation of cardiac wall motion and excursion of AV valves, followed instantaneously by the appearance of coagulation in all the other chambers (Figure 3). The AV valves appeared to be tethering with a fixed gap in mid annular-position, and an expanding rapid appearance of pleural effusion was appreciated at the end (Figure 4).

**Follow-up and outcomes:** the patient died during the ACLS interventions.

**Informed consent:** this case study is completely anonymized to protect the identity of the patient; thus, a formal informed consent was not obtained from this deceased patients.

## Discussion

Point of care echocardiogram is sometimes performed during the pulse check periods of CPR attempts to investigate the conditions possibly impacting on electromechanical dissociation (EMD) or PEA. Potential findings such as cardiac tamponade, tension pneumothorax, and low volume state can be rapidly resolved while other complicated catastrophic conditions such as cardiac rupture, aortic dissection, and flail valve or low cardiac output state can be quickly recognized to drive emergent and appropriate measures. Early recognition of reversible and actionable findings can sometimes provide favorable outcomes. In this case, we did not find any culpable and reversible structural abnormalities for this patient´s condition besides a toxic metabolic cardiac arrest from hyperkalemia, which was complicated by severe metabolic acidosis and sinoventricular rhythm (Figure 1A-C). Sinoventricular rhythm is a rare but pathognomonic arrhythmia seen in severe hyperkalemia and metabolic acidosis [1,2]. However, we were able to fortuitously obtain the sequence of echocardiographic events occurring moments before cardiac standstill. Clip # 54 in Figure 3 shows the beginning of spontaneous echo contrast in the left ventricle progressing into the rest of the cardiac chambers. This provides a rare opportunity to capture the images of the dying heart in real time. An investigation of the literature reveals a paucity of neither information nor detailed illustrations on the echocardiographic findings of a dying heart. Few images can be found showing thrombus in the cardiac chambers of a still heart. However, we cannot find a publication or report that catalogues and illustrate the sequence of echo findings leading up to the last heartbeat.

SEC can be associated with cardiac hypokinesis and stasis [3-5]. Hemostasis can be seen in the left atrial appendage of patients with atrial fibrillation and portends a risk for thrombus formation [6]. Whereas SEC can be seen in the left atrium in atrial fibrillation, it can also be seen in the ventricular chambers when there is significant enlargement or cardiac hypokinesis. In some cases, in situ cardiac thrombus is noted on routine echo done on patients with suspected heart failure and cardiomyopathy. In general, thrombus formation is believed to occur over a period following cardiac hypokinesis and hemostasis. However, in this case, all chambers were noted with real time development of intracardiac coagulation. Cardiac standstill coincides with complete loss of electrical activity and instantaneous appearance of amorphous echo-dense material in the cardiac chambers like thrombus. This observation was previously reported in human dying hearts and confirmed as rapidly formed red thrombi in canine models [5]. The rapid and concurrent observation of blood stasis and clot formation suggests enhanced interplay of stasis and dysregulated coagulation cascade during death. We term this phenomenon, ‘Hemostatic Instantaneous Coagulation on Echo´ (HICE). It is unknown if certain agents administered during resuscitation especially calcium compounds can precipitate or influence the development of HICE. Further studies may be warranted to investigate this possibility. HICE may be the defining feature of the final heartbeat. Epinephrine and defibrillation shocks are sometimes applied to patients found down with asystole, and often in futility [7]. The occurrence of HICE may represent widespread coagulation which could affect the viability of organs and overall survival.

## Conclusion

In this case, the arrhythmia eventually degenerated into asystole that was associated with hemostasis and rapidly progressive coagulation from LV chamber to all the other cardiac chambers. The last heartbeats were associated with a hyperacute development of hemostatic coagulation in the cardiac chambers. We illustrate and describe these findings that have not been previously characterized in appreciable detail. We refer summarily to the echocardiographic findings of the last heartbeat in this patient with cardiac arrest as ‘Hemostatic Instantaneous Coagulation on Echo´ (HICE). The finding of HICE could portend poor prognosis with resuscitative efforts and could guide decisions about performing interventions that may be futile. Increasing the awareness about this phenomenon could lead to better understanding, novel ideas and approaches in cardiac resuscitation science.
